# Prevalence and diversity of *Bartonella* species in small rodents from coastal and continental areas

**DOI:** 10.1038/s41598-019-48715-y

**Published:** 2019-08-26

**Authors:** Dalytė Mardosaitė-Busaitienė, Jana Radzijevskaja, Linas Balčiauskas, Maksim Bratchikov, Vaclovas Jurgelevičius, Algimantas Paulauskas

**Affiliations:** 1 Faculty of Natural Sciences, Vytautas Magnus University, Vileikos str. 8, LT- 44404 Kaunas, Lithuania; 2 Laboratory of Mammalian Ecology, Nature Research Centre, Akademijos st. 2, LT-08412 Vilnius, Lithuania; 3 Department of Physiology, Biochemistry, Microbiology and Laboratory Medicine, Institute of Biomedical Sciences, Faculty of Medicine, Vilnius University, M.K. Čiurlionio g. 21/27, LT-03101 Vilnius, Lithuania

**Keywords:** Microbial genetics, Pathogens

## Abstract

Worldwide, *Bartonella* infections are known to inflict a wide range of mammals and, within rodents alone, more than 20 *Bartonella* species have been detected. There is, however, a lack of studies on the presence of *Bartonella* spp. in rodents in the Baltic region. We analysed 580 individuals belonging to eight small rodent species trapped in coastal and continental areas of Lithuania during 2015–2016. The presence of *Bartonella* DNA was examined by real-time PCR targeting the *ssrA* gene. The molecular characterization of the bacteria strains was based on sequence analysis of two housekeeping genes (*rpoB*, *groEL*) and the intergenic spacer region (ITS). For the rodents overall, the prevalence of *Bartonella* spp. was 54.8%, while the prevalence figures for each of the individual species were 8.3% in *M*. *musculus*, 15.8% in *A*. *agrarius*, 33.3% in *M*. *arvalis*, 42.4% in *M*. *glareolus*, 53.4% in *M*. *oeconomus*, 57.5% in *M*. *minutus*, 79.6% in *A*. *flavicollis* to 80% in *M*. *agrestis*. Sequence analysis revealed that the *Bartonella* strains belonged to the *B*. *grahamii*, *B*. *taylorii*, *B*. *rochalimae*, *B*. *tribocorum*, *B*. *coopersplainsensis* and *B*. *doshiae* genogroups. The highest *Bartonella* infection rates and the highest species diversity were both detected in rodents captured in the coastal area. To our knowledge, these are the first reports of the presence of *B*. *coopersplainsensis*, *B*. *doshiae* and *B*. *tribocorum* in Lithuania.

## Introduction

Bartonellae are facultative intracellular, fastidious, gram-negative bacteria that are transmitted to humans and other mammals by bloodsucking arthropod vectors such as fleas, mites, sand fleas and ticks^1,2^. The most important vectors for the maintenance and transmission routes of the *Bartonella* species are fleas, that feed on mammalian hosts such as cats, dogs, rodents, insectivores and rabbits^3^. There are currently 35 *Bartonella* species and three subspecies with standing in the Taxonomy Database of the National Center for Biotechnology Information (http://www.bacterio.net/Bartonella.html). However, the number of species in genus *Bartonella* is growing, as not all *Bartonella* species have been validated. Currently, 45 official and candidate *Bartonella* species have been detected in vertebrates, and at least fifteen of them have been related to human diseases^4^. During their evolution, *Bartonella* spp. have adapted to a variety of reservoir hosts and have become pathogenic when introduced into an incidental host^5,6^. With more than 20 *Bartonella* species associated with rodents, this group of mammals represents an important group of potential reservoirs for many *Bartonella* infections that have been reported worldwide^7^. Human pathogenic *Bartonella* species such as *B*. *elizabethae*, *B*. *tribocorum*, *B*. *grahamii*, *B*. *rochalimae*, *B*. *vinsonii* and *B*. *washoensis* have been isolated from various rodent species^3,5,7^. More than one *Bartonella* species can circulate in rodent communities, and multiple *Bartonella* genotypes in the same rodent host have been reported^8,9^.

The yellow-necked mouse (*Apodemus flavicollis*), wood mouse (*Apodemus sylvaticus*), striped field mouse (*Apodemus agrarius*), bank vole (*Myodes glareolus*), common vole (*Microtis arvalis*) field vole (*Microtus agrestis*) and root vole (*Microtus oeconomus*) are rodent species that play important roles in the maintenance and circulation of *Bartonella* infections in Europe^5,8^. *Bartonella* pathogens have been reported in several rodent populations in Poland, Germany, Denmark and Sweden (reviewed by Gutierrez *et al*.^8^). However, *Bartonella* epidemiology and host-pathogen associations in Europe, including in the Baltic region, are insufficiently characterized.

The objectives of this study were to investigate the prevalence of *Bartonella* species in different species of rodents collected from coastal and continental areas of Lithuania using real-time PCR targeting the *ssrA* gene, and to characterize the genetic diversity of *Bartonella* strains by sequence analysis of two housekeeping genes (*rpoB*, *groEL*) and the 16S-23S rRNA intergenic species region (ITS).

## Results

### Prevalence of *Bartonella* species in rodents

Based on real-time PCR analysis, a total of 318 of the rodent DNA samples (54.8% of the 580 samples analysed) were found positive for *Bartonella* spp., and out of these, 127 samples had C_t_ value below 30.

The highest prevalence of *Bartonella* spp. was found in *A*. *flavicollis* and *M*. *agrestis* (almost 80%); in *M*. *minutus*, *M*. *glareolus* and *M*. *oeconomus* the prevalence was in the range of 30–50%, while in other rodents, the prevalence was lower (Table 1).

**Table 1:** Prevalence of *Bartonella* spp. in different small rodent species from Lithuania, 2015–2016.

Region			No	Location	Habitat	Coordinates		*Bartonella* spp. in rodents, n/N (%)								
								*A*. *fla*	*A*. *agr*	*M*. *min*	*M*. *mus*	*M*. *gla*	*M*. *oec*	*M*. *arv*	*M*.*agr*	Total
Western Lithuania	Coastal area	Curonian Spit	1	Amber gulf	meadow	55°33′06.0″N	21°07′31.5″E	15/15 (100)	—	5/10 (50)	—	1/2	1/1	1/2	—	23/30(76.7)
			2	Juodkrante	ecotone	55°32′30.9″N	21°07′02.4″E	78/89 (87.6)	—	—	—	13/26 (50)	0/2		—	91/117(77.8)
			3	Pervalkos gulf	meadow	55°24′37.7″N	21°05′08.4″E	15/22 (68.2)	—	14/24 (58.3)	—	1/3	—	—	—	30/49(61.2)
			4	Karvaiciai gulf	meadow	55°23′15.4″N	21°04′19.4″E	21/25 (84)	—	1/1	—	—	0/13	—	—	22/39(56.4)
			5	LybisCape	meadow	55°16′57.1″N	20°57′30.8″E	15/17 (88.2)	—	1/1	—	0/2	—	0/1	—	16/21(76.2)
							Subtotal	144/168 (85.7)	—	21/36 (58.3)	—	15/33 (45.5)	1/16 (6.3)	1/3 (33.3)	—	182/256(71.1)
		Nemunas River Delta	6	Rusne	flooded meadows	55°19′26.23″N	21°20′24.15″E	—	4/52 (7.7)	2/3 (66.7)	—	4/19 (21.1)	28/40 (70)	—	12/12(100)	50/126(39.7)
			7	Zalgiriai	flooded forest	55°18′40.0″N	21°26′10.0″E	2/5	1/7	0/1	—	1/14 (7.1)	2/2	—	6/9	12/38(31.6)
							Subtotal	2/5 (40)	5/59 (8.5)	2/4 (50)	—	5/33 (15.2)	30/42 (71.4)	—	18/21(85.7)	62/164(43.7)
Eastern Lithuania	Continentalarea		8	Lukstas	deciduous forest in peninsula	55°51′0.94″N	26°12′6.11″E	1/2	5/7	—	—	29/47 (61.7)	—	—	─	35/56(62.5)
			9	Beistrakiai	ecotone	54°54′22.3″N	24°20′28.6″E	9/17 (52.9)	2/10 (20)	—	1/12 (8.3)	—	—	—	2/4	14/43(32.6)
			10	Dubingiai	mixed forest	55°03′38.1″N	25°27′03.7″E	1/3	—	—	—	1/11 (9.1)	—	—	—	2/14(14.3)
			11	Varniskes	mixed forest	54°58′00.9″N	25°22′40.6"E	3/6	—	—	—	1/14 (7.1)	—	—	—	4/20(20)
			12	Elektrenai	deciduous forest in island	54°45′37.22″N	24°40′41.45″E	—	—	—	—	19/27 (70.4)	—	—	—	19/27(70.4)
							Subtotal	14/28 (50)	7/17 (41.2)	—	1/12 (8.3)	50/99 (50.5)	—	—	2/4 50)	74/160(46.3)
							Total	160/201 (79.6)	12/76 (15.8)	23/40 (57.5)	1/12 (8.3)	70/165 (42.4)	31/58 (53.4)	1/3 (33.3)	20/25(80)	318/580(54.8)

*Abbreviations*: N, number of individuals tested; n, number of individuals infected; *A*. *fla*, *Apodemus flavicollis*; *A*. *agr*, *Apodemus agrarius*; *M*. *min*, *Micromys minutus*; *M*.*mus*, *Mus musculus*; *M*. *gla*, *Myodes glareolus*; *M*. *oec*, *Microtus oeconomus*; *M*. *arv*, *Microtus arvalis*; *M*. *agr*, *Microtus agrestis*.

*Bartonella*-infected *A*. *flavicollis*, *A*. *agrarius* and *M*. *agrestis* were found in all locations where these rodents were captured with the prevalence of infection ranging in different locations from 33.3 to 100%, from 7.7 to 71.4%, and from 50 to 100% respectively (Table 1). *Bartonella*-infected *M*. *glareolus* were found in nine of the 10 sampling locations: with the infection prevalence ranging from 0 to 70.4%. *M*. *minutus* and *M*. *oeconomus* were trapped only in the coastal area of the western part of the country, with the overall prevalence of infection estimated at 58.3% on the Curonian Spit and 50% in the Nemunas River Delta in *M*. *minutus* and 6.3% and 71.4% for the respective locations in *M*. *oeconomus* (Table 1). One *Bartonella*-infected *M*. *musculus* specimen was found in a mixed forest-meadow ecotone in the continental area (site 9) (Table 1).

Comparing *A*. *flavicollis* across the country, significantly higher overall *Bartonella* infection rates (85.7%; 144/168) were detected in those trapped on the Curonian Spit (odds ratio, 1.9; 95% confidence interval, 2.845–14.301; p < 0.000). Likewise, among all investigated areas, a significantly higher overall prevalence of *Bartonella* was observed on the Curonian Spit (71.1%, 182/256; odds ratio, 1.2; 95% confidence interval, 2.346–4.720; p < 0.000) with the highest prevalence of *Bartonella* detected in sampling site 2 (77.8%, 91/117; odds ratio, 1.3; 95% confidence interval, 2.265–5.825; p < 0.000).

### Diversity of *Bartonella* species in rodents

Eighty-seven *Bartonella*-positive PCR products of partial *rpoB*, *groEL* genes and ITS region derived from 56 different rodent specimens of eight species were subjected to sequence analysis (Table 2). A total of 73 good-quality sequences of *rpoB* (n = 43), *groEL* (n = 9) genes, and ITS region (n = 21) were obtained and analyzed.

**Table 2:** Distribution of *Bartonella* species in the rodent communities in Lithuania.

Species	n	B. grahamii	B. taylorii	B. rochalimae-like	B. tribocorum	B. coopersplainsensis	B. doshiae
**Curonian Spit Site 1–5**							
*A. flavicollis*	11	2	9	0	0	0	0
*M. minutus*	2	2	0	0	0	0	0
*M. glareolus*	6	3	2	1	0	0	0
*M. agrestis*	1	1	0	0	0	0	0
*M. arvalis*	1	1	0	0	0	0	0
**Nemunas River Delta Site 6–7**							
*A. agrarius*	3	1	0	0	1	1	0
*M. minutus*	1	1	0	0	0	0	0
*M. glareolus*	2	0	2	0	0	0	0
*M. oeconomus*	6	1	4	1	0	0	0
*M. agrestis*	5	0	4	0	0	0	1
**Continental part of country Site 8–12**							
*A. flavicollis*	2	1	1	0	0	0	0
*A. agrarius*	4	3	0	0	1	0	0
*M. musculus*	1	1	0	0	0	0	0
*M. glareolus*	9	2	7	0	0	0	0
*M. agrestis*	2	0	2	0	0	0	0

n – number of infected rodents included in sequence analysis.

**Figure 1:**
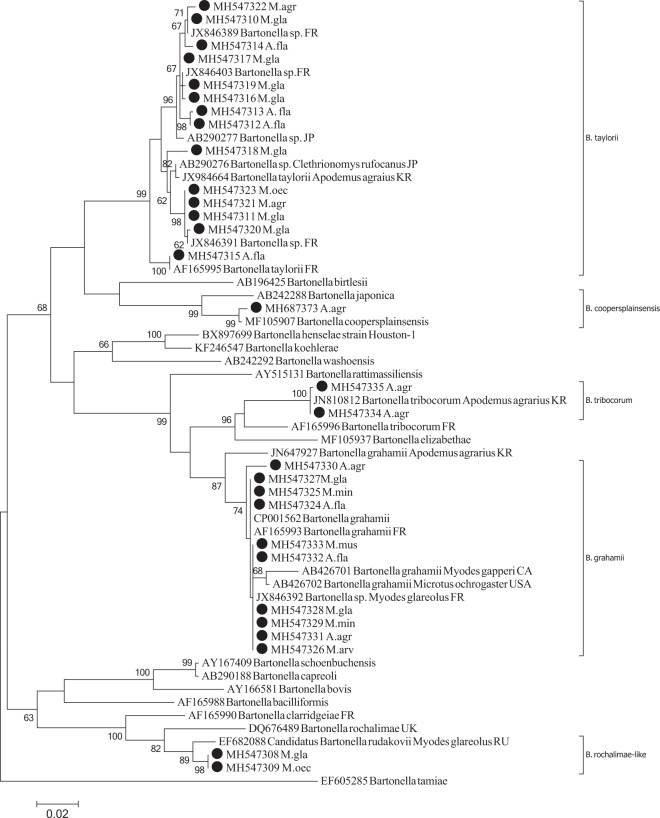
Maximum-likelihood phylogenetic tree for the partial *rpoB* gene of *Bartonella* spp. The phylogenetic tree was created using the Tamura-Nei model and bootstrap analysis of 1000 replicates. Identification source (rodent host) is given after the code of sample. Sequences MH547313, MH547314 and MH547315 are representative of three other samples sequenced in this study (all derived from *A*. *flavicollis*); Sequence MH547320 is representative of one other sample derived from *M*. *glareolus;* Sequence MH547321 is representative of two other samples (all derived from *M*. *agrestis*); Sequence MH547327 is representative of other sample from *M*. *glareolus;* Sequence MH547329 is representative of other sample from *M*. *minutus*; Sequence MH547328 are representative of two other samples sequenced in this study (all derived from *M*. *glareolus*). Samples sequenced in the present study are marked. Abbreviations: A. fla – *Apodemus flavicollis*; M. min – *Micromys minutus*; M. gla – *Myodes glareolus*; A. agr – *Apodemus agrarius*; M. mus – *Mus musculus*; M. oec. – *Microtus oeconomus*; M. agr – *Microtus agrestis*.

Analysis of 43 partial *rpoB* gene sequences showed that the *Bartonella* species circulating among the investigated rodents were heterogenic and belonged to *B*. *grahamii*, *B*. *taylorii*, *B*. *tribocorum*, *B*. *coopersplainsensis* and *B*. *rochalimae* genogroup (Fig. 1). *Bartonella* strains derived from six rodent species *A*. *flavicollis* (n = 2), *A*. *agrarius* (n = 3), harvest mouse *Micromys minutus* (n = 3), common house mouse *Mus musculus* (n = 1), *M*. *glareolus* (n = 5) and *M*. *arvalis* (n = 1) were 95–100% similar to *B*. *grahamii* sequences deposited in the GenBank (CP001562, JN810824, JN647927) (Fig. 1). Three *B*. *grahamii* genotypes (differing at 5 nucleotides positions) were identified (Fig. 1). *Bartonella rpoB* sequences derived from four species of rodents *A*. *flavicollis* (n = 7), *M*. *glareolus* (n = 11), *M*. *agrestis* (n = 4) and *M*. *oeconomus* (n = 1) were 97–100% identical (differing at 48 nucleotide positions) to *B*. *taylorii* sequences obtained in the GenBank (AF165995; JX984664) (Fig. 1). Twelve different *B*. *taylorii* genotypes among Lithuanian isolates were detected (Fig. 1): seven genotypes in *M*. *glareolus* (differing at 29 nucleotide positions); four in *A*. *flavicollis* (differing at 28 nucleotide positions) and two in *M*. *agrestis* (differing at 10 nucleotide positions). Two *Bartonella rpoB* sequences derived from *A*. *agrarius* (sites 6, 8) shared 99–100% identity to the *B*. *tribocorum* detected in *A*. *agrarius* from South Korea (GenBank: JN81812) (Table 2; Fig. 1). *Bartonella* sequences derived from *M*. *glareolus* (site 2), *M*. *oeconomus* (site 7) and *A*. *agrarius* (site 6) were 98–99% identical to *B*. *rochalimae* (GenBank: DQ676489), *Candidatus Bartonella rudakovii* (GenBank: EF682088) and *B*. *coopersplainsensis* (detected in rats from Thailand, GenBank: MF105907) respectively (Fig. 1).

**Figure 2:**
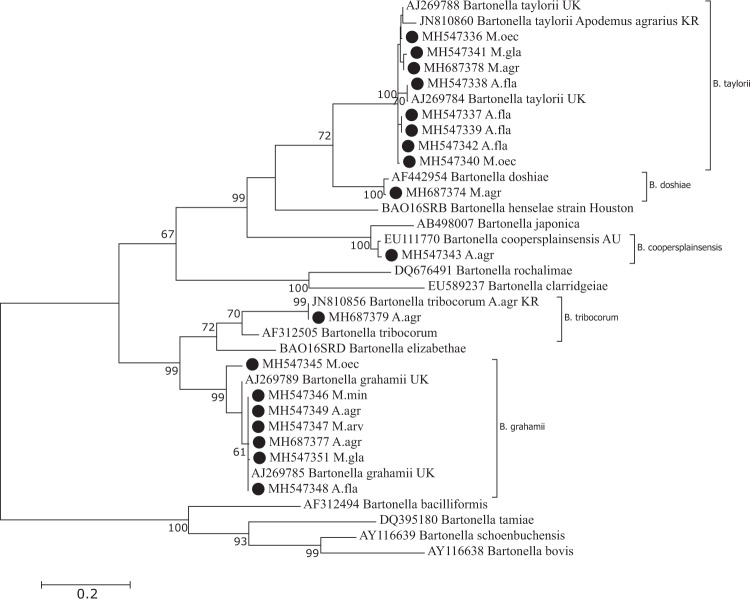
Maximum-likelihood phylogenetic tree for the partial ITS region of *Bartonella* spp. The phylogenetic tree was created using the Tamura-Nei model and bootstrap analysis of 1000 replicates. Identification source (rodent host) is given after the code of sample. Sequence MH547346 is representative of three other samples sequenced in this study (all derived from *M*. *minutus*); Sequence MH547348 is representative of one other sample derived from *A*. *flavicollis*; Samples sequenced in the present study are marked. Abbreviations: A. fla – *Apodemus flavicollis*; M. min – *Micromys minutus*; M. gla – *Myodes glareolus*; A. agr – *Apodemus agrarius*; M. mus – *Mus musculus*; M. oec. – *Microtus oeconomus*; M. agr – *Microtus agrestis*.

The sequence analysis of the partial ITS region of 21 samples revealed the presence of *Bartonella* strains from the *B*. *grahamii*, *B*. *taylorii*, *B*. *tribocorum*, *B*. *coopersplainsensis* and *B*. *doshiae* genogroup (Fig. 2). Ten *Bartonella* strains derived from six rodent species *A*. *flavicollis* (n = 2), *A*. *agrarius* (n = 2), *M*. *minutus* (n = 3), *M*. *arvalis* (n = 1), *M*. *oeconomus* (n = 1) and *M*. *glareolus* (n = 1) were 97–100% identical (differing at 17 nucleotide positions) to *B*. *grahamii* sequences deposited in the GenBank (AJ269785; AJ269789). Four *B*. *grahamii* genotypes were identified (Fig. 2). Eight *Bartonella* ITS region sequences derived from four rodent species *A*. *flavicollis* (n = 4), *M*. *oeconomus* (n = 2), *M*. *agrestis* (n = 1) and *M*. *glareolus* (n = 1) shared 97–99% similarity with *B*. *taylorii* sequences deposited in the GenBank (JN10860; AJ269784). Obtained sequences were heterogenic (differing at 33 nucleotide positions), and eight *B*. *taylorii* genotypes were detected (Fig. 2). The *Bartonella* ITS region sequence derived from *A*. *agrarius* (site 6) was 99% identical (differing at nine nucleotide positions) to *B*. *coopersplainsensis* detected in rats from the Australia (GenBank: EU111770). Two sequences, one derived from *A*. *agrarius* and another from *M*. *agrestis* (site 6) were 98–99% identical to *B*. *tribocorum* (GenBank: JN810856) and *B*. *doshiae* (GenBank: AF442954) respectively (Fig. 2).

**Figure 3:**
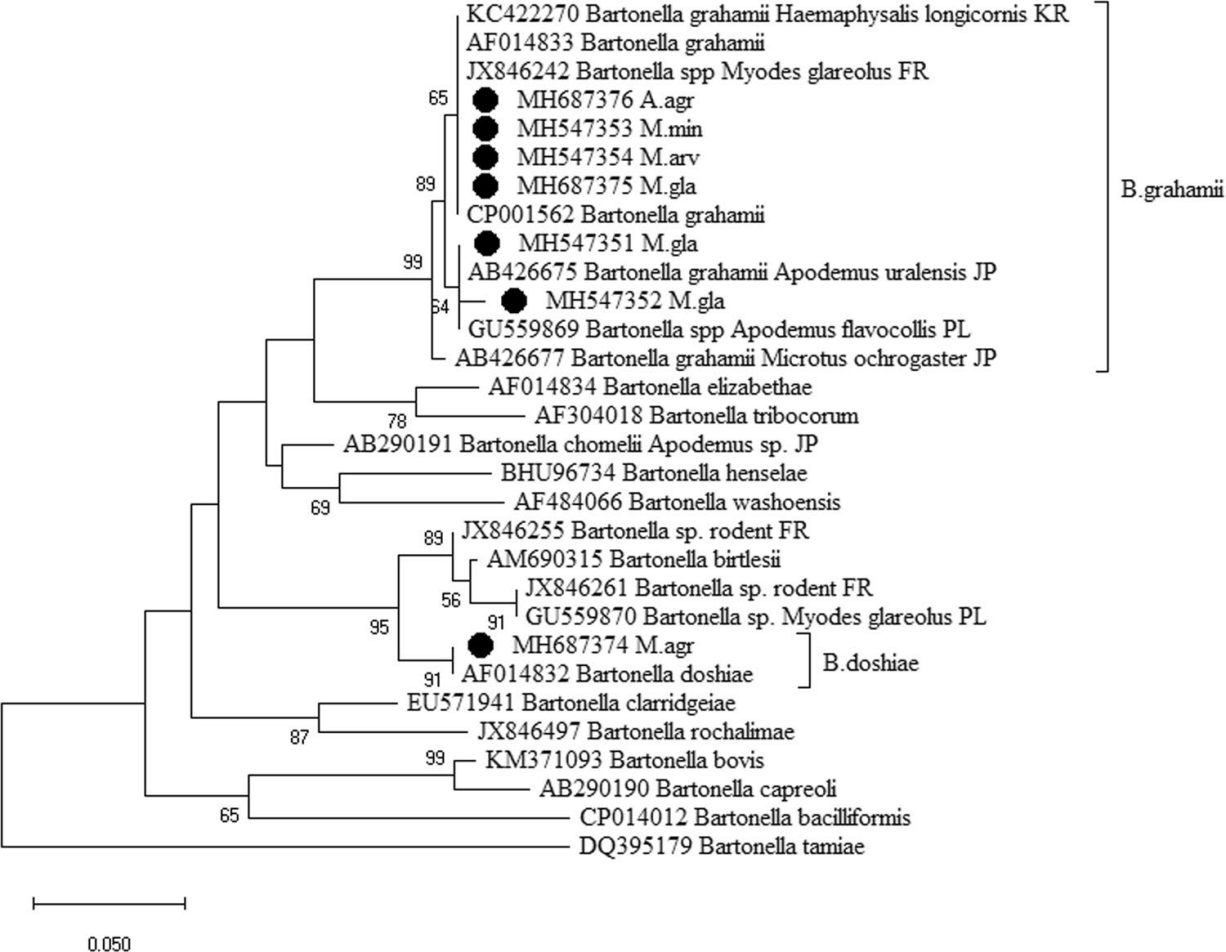
Maximum-likelihood phylogenetic tree for the partial *groEL* gene of *Bartonella* spp. The phylogenetic tree was created using the Tamura-Nei model and bootstrap analysis of 1000 replicates. Identification source (rodent host) is given after the code of sample. Samples sequenced in the present study are marked. Sequence MH547353 is representative of other sample from *M*. *minutus*. Abbreviations: M. min – *Micromys minutus*; M. gla – *Myodes glareolus*; A. agr – *Apodemus agrarius*; M. arv – *Microtus arvalis*; M. agr – *Microtus agrestis*.

The *groEL* gene sequences showed 97–100% similarity to *B*. *grahamii* sequences deposited in the GenBank (AB426677; AF014833; GU559869) (Fig. 3). Phylogenetic analysis demonstrated the presence of three *B*. *grahamii* genotypes in *M*. *glareolus*, (n = 3), *M*. *minutus* (n = 2), *A*. *agrarius* (n = 1), *M*. *arvalis* (n = 1) and *M*. *agrestis* (n = 1). One *Bartonella groEL* sequence derived from *M*. *agrestis* (site 6) was 100% identical to *B*. *doshiae* (GenBank: AF014832) (Fig. 3).

## Discussion

The present study represents the prevalence and molecular characterization of the *Bartonella* strains circulating in rodent communities in the continental and coastal areas of Lithuania. Phylogenetic analysis based on two housekeeping genes and ITS region demonstrated that small rodents in Lithuania harbor multiple *Bartonella* species belonging to six genogroups, specifically *B*. *grahamii*, *B*. *taylorii*, *B*. *tribocorum*, *B*. *coopersplainsensis*, *B*. *doshiae* and *B*. *rochalimae* (Table 2).

Worldwide, the prevalence of *Bartonella* spp. is ranging from 25 to 80%. The high rate of *Bartonella* infection in rodent communities suggests a reciprocal adaptation between the bacteria and their reservoirs^8^. We found the overall prevalence of *Bartonella* species in different rodent species in Lithuania ranging from 8.3 to 80% with the highest *Bartonella*-infection rates in *M*. *agrestis* (80%) and *A*. *flavicollis* (79.6%) (Table 1). Our findings are similar to those reported in *A*. *flavicollis* from the eastern Germany (84.4%^10^) but higher than those from Slovakia (63.0%; 244/387^11^), Slovenia (62.7%; 27/43^12^), Denmark (53.3%; 8/15^13^), Poland (42.2; 68/161^14^). In *M*. *agrestis*, lower infection rates were found in Sweden (33.3%; 1/3^15^), Denmark (33.3%; 5/15^12^) and France (11%; 1/9^5^). In Lithuania, a high *Bartonella* prevalence was also detected in *M*. *minutus* (57.5%), *M*. *oeconomus* (53.4%) and *M*. *glareolus* (42.4%). In other European countries, reported prevalence of *Bartonella* infection in *M*. *glareolus* include 69.0% (160/232) in Slovakia, 56.4% (252/447) in France, 52.8% (21/36) in Germany and 15.0% (9/60) in Sweden^10,11,15,16^. *Bartonella*-infected *M*. *oeconomus* have been found in Poland with an 11.1% (14/128) prevalence of infection^14^, a figure that s five times lower than that obtained in our study. The lowest overall prevalences of *Bartonella* spp. in the present study were found in *A*. *agrarius* (15.8%) and *M*. *musculus* (8.3%). In comparison, studies conducted in Slovenia and Slovakia detected 26.6% (8/30) and 9% (31/344) prevalence of *Bartonella* spp. in *A*. *agrarius*^11,12^, while Holmberg *et al*.^15^ reported a 5.6% (1/18) prevalence of infection in *M*. *musculus* from Sweden.

The observed differences in the prevalence of *Bartonella* spp. might be related to habitats and to the abundance of the rodent species^16^. In this study, extremely high *Bartonella*-infection rates (56.4–77.8%) were detected in rodent communities on the Curonian Spit (Table 1), a region characterized by high diversity of natural habitats this resulting in diversity in animal and plant species. The dominant rodent species in all habitats on the Curonian Spit was *A*. *flavicollis*, this rodent species characterized by high *Bartonella*-infection rates (*B*. *grahamii* and *B*. *taylorii* most frequent) (Tables 1, 2).

In Europe, *B*. *grahamii* and *B*. *taylorii* have been detected in *A*. *flavicollis*, *A*. *agrarius*, *A*. *sylvaticus*, herb field mouse (*Apodemus uralensis*), steppe field mouse (*Apodemus witherbyi*), *M*. *minutus*, *M*. *glareolus*, *M*. *arvalis*, *M*. *agrestis* and *M*. *musculus*; while *B*. *rochalimae* has been detected in *A*. *flavicollis*, *M*. *glareolus and M*. *arvalis* (reviewed by Špitalska *et al*.^11^), and *B*. *doshiae*, in *A*. *flavicollis*, *A*. *agrarius*, *M*. *agrestis* and *M*. *glareolus* (reviewed by Buffet *et al*.^17^).

In this study, the 52.9% of sequences derived from small rodents were ascribable to *B*. *taylorii*. In general, *B*. *taylorii* strains demonstrate high diversity and are frequently found in mice, as well as in *Myodes* and *Microtus* voles, inhabiting boreal forests of the Eurasian continent (reviewed by Buffet *et al*.^5^). In line with this, the present study showed high diversity of *B*. *taylorii* strains in Lithuanian rodents. Phylogenetic analysis of *rpoB* gene revealed the presence of twelve *B*. *taylorii* genotypes associated with *A*. *flavicollis* mice (four out of twelve) and three species of voles *M*. *glareolus*, *M*. *agrestis* and *M*. *oeconomus* (eight out of twelve) (Fig. 1). The high variability of *B*. *taylorii* strains in rodents could be explained by a potential accelerated evolution of the *Bartonella* genus in small rodents as a result of frequent recombination events, horizontal gene acquisitions, and accumulation of mutations (reviewed by Gutierrez *et al*.^8^).

In this study, human pathogenic *B*. *grahamii* strains were detected in 39.2% (20 out of 51) of rodent specimens and showed lower sequence diversity in comparison to *B*. *taylorii*. *B*. *grahamii* was detected in four species of mice (*A*. *flavicollis*, *A*. *agrarius*, *M*. *musculus*, *M*. *minutus*), and four species of voles (*M*. *glareolus*, *M*. *oeconomus*, *M*. *arvalis* and *M*. *agrestis*) (Table 2). Similar results have been observed in some European countries, while higher polymorphism of *B*. *grahamii* has been observed in Asia^17^. The low diversity of *B*. *grahamii* has been explained by the spread of these bacteria from Asia to Europe by the introduction of its hosts and/ or a severe bottleneck relatively with too little time having elapsed for polymorphisms to reaccumulate^17^.

*B*. *rochalimae* is typically associated with carnivores (cats, dogs, foxes and raccoons), and Eremeeva *et al*.^18^ reported clinical case of bacteremia, fever, and splenomegaly in a patient who traveled to Peru. In Europe, *Bartonella* spp. related to *B*. *rochalimae* group have been detected in *A*. *agrarius*, *A*. *flavicollis* and *M*. *arvalis* from Slovakia^11,19^. We report *Bartonella* spp. from *B*. *rochalimae* group in *M*. *oeconomus* for the first time (Fig. 1).

The main vectors for the maintenance and transmission of *B*. *grahamii*, *B*. *taylorii* and *B*. *rochalimae* among populations of small mammals are fleas^5^, these also having been identified as a risk factor for the transmission of *Bartonella* pathogens to humans. In Lithuania, strains identical or similar to *B*. *grahamii*, *B*. *taylorii* and *B*. *rochalimae* were detected in five flea species with an overall prevalence of 29.1%. *B*. *grahamii* was also detected in *Ixodes ricinus* ticks^20^.

This study is the first report of *B*. *doshiae* infection in *M*. *agrestis* from Lithuania. In Europe, this species has been found with very low prevalence and genetic diversity in *Apodemus* spp., *M*. *glareolus* and *M*. *agrestis*^5^.

Previous studies strongly supported the association of *B*. *tribocorum* with rats of the genus *Rattus*^5^. However, Ko *et al*.^21^ detected *B*. *tribocorum* in *A*. *agrarius* in South Korea. In the current study, the human pathogenic *B*. *tribocorum* was identified in *A*. *agrarius* in coastal (site 6) and continental (site 8) areas with 100% similarity to the *B*. *tribocorum* isolate from South Korea (Fig. 1). Kraljik *et al*.^19^ reported 94.8% similarity of *Bartonella* isolates derived from *A*. *agrarius* to the *B*. *elizabethae*/*B*. *tribocorum* clade. Our study is the first detection of *B*. *coopersplainsensis* in *A*. *agrarius* in the coastal area of the Nemunas River Delta. This result is unexpected, as *B*. *coopersplainsensis* is distributed in Asia and Australia, and the species spread in Europe is associated with rats. The detection of *B*. *tribocorum* and *B*. *coopersplainsensis* in *A*. *agrarius* could be explained through the accidental contact between *A*. *agrarius* and rats in agricultural habitats.

The highest *Bartonella* species diversity was detected in the specific habitats of flooded meadow and flooded forest within the Nemunas River Delta: five rodent species trapped in this area harbored six different *Bartonella* species (Table 2). The high *Bartonella* species diversity detected in the Nemunas River Delta could be explained by another particular features of this area, namely that it is the most important stopover area for migratory birds in Lithuania, with about 200 species also breeding^22^. Migratory birds are important carriers for a variety of ectoparasites with a great potential to assist in their spread. An alternative explanation for the high *Bartonella* species diversity in the Nemunas Delta is that the area is also characterized by a high intensity of local shipping, thereby also providing a suitable environment for rats and consequently for the transmission of rat-related *Bartonella* spp.

In conclusion, the present study demonstrates that small rodents from both coastal and continental areas of Lithuania are frequently infected with *Bartonella* spp. Our findings provide evidence of a high diversity of *Bartonella* species and the presence of strains identical or closely related to the humans pathogenic *B*. *grahamii*, *B*. *tribocorum*, and *B*. *rochalimae*. This study provides the first detection of *B*. *coopersplainsensis*, *B*. *doshiae* and *B*. *tribocorum* in Lithuania. To our knowledge, it is also the first report of *Bartonella* spp. in the agriculture-related rodent species *A*. *agrarius* and indoor rodent species such as *M*. *musculus* in Lithuania.

## Material and Methods

### Ethical statement

Rodents sampling was conducted with permission from the Environmental Protection Agency (EPA) and approved by the Ministry of Environment of the Republic of Lithuania, licenses No. 22 (2015-04-10) and No. 12 (2016-03-30) in accordance with Lithuanian (the Republic of Lithuania Law on Welfare and Protection of Animals No. XI-2271) and European legislation (Directive 2010/63/EU) on the protection of animals. Live traps were checked at least twice a day. Captured animals were anesthetized with CO_2_ exposure and killed humanely according to the Lithuanian Animal Protection Act.

### Rodents trapping

**Figure 4:**
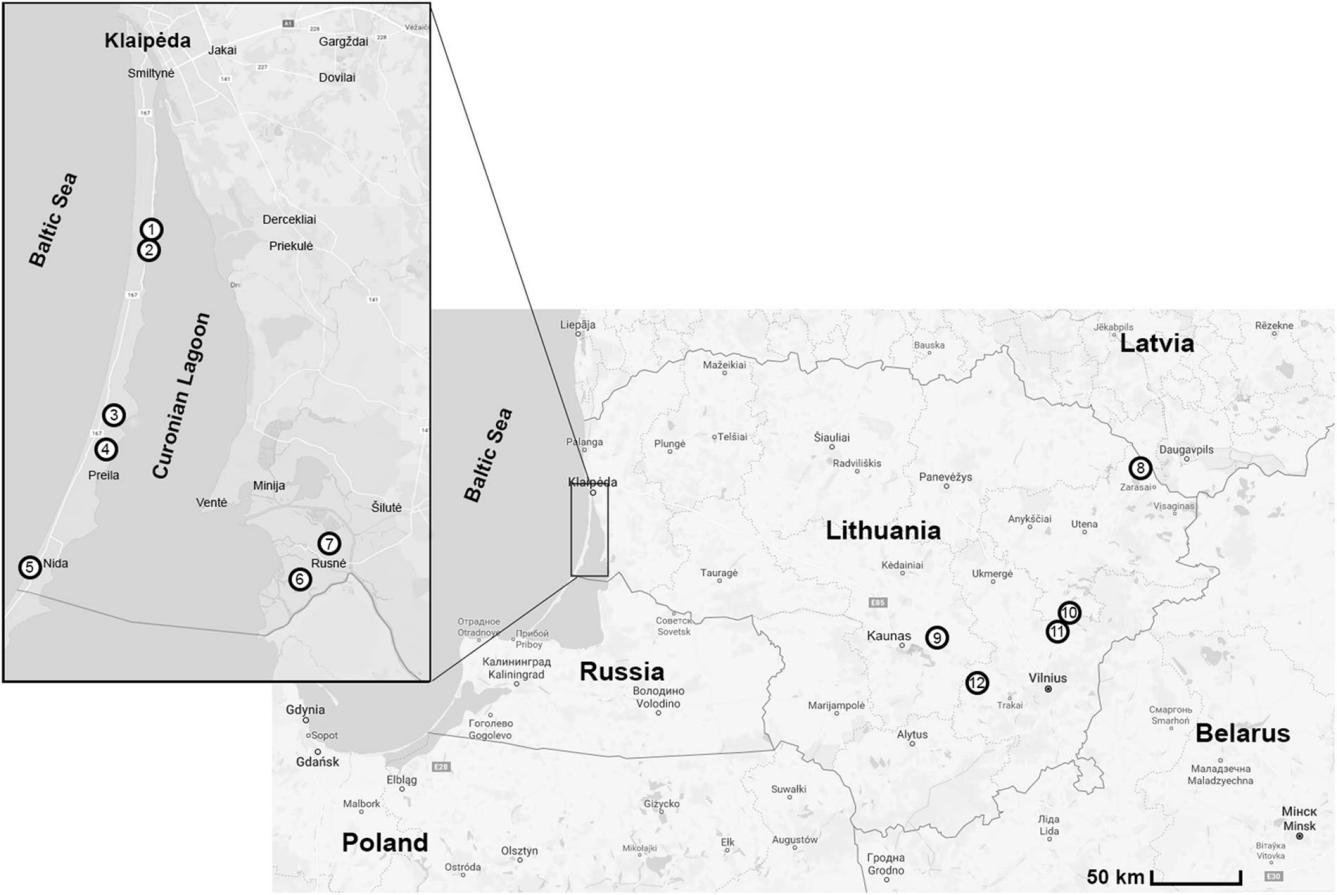
Small rodent trapping sites on the Curonian Spit (sites 1–5), the Nemunas River Delta (sites 6–7) and continental (sites 8–12) areas of Lithuania, 2015–2016.

A total of 580 rodents representing eight species – *A*. *flavicollis*, *A*. *agrarius*, *M*. *minutus*, *M*. *musculus*, *M*. *glareolus*, *M*. *oeconomus*, *M*. *agrestis* and *M*. *arvalis* were trapped with live-traps or snap traps in 12 locations situated in western (coastal area; sites 1–7) and eastern (continental area; sites 8–12) parts of Lithuania during 2015–2016 (Fig. 4; Table 1).

In the western part of the country rodents were trapped on the Curonian Spit (sites 1–5, close to the Baltic Sea) and in the Nemunas River Delta (sites 6–7). The Curonian Spit is a narrow sand peninsula (2 km wide and 98 km long, half of which is in Lithuania) in the southeastern coast of the Baltic Sea separating the Curonian Lagoon from the Baltic Sea. Rodent sampling on the Curonian Spit was conducted in coastal meadows and ecotone between meadow and mixed forest. In the Nemunas River Delta, trapping was conducted in two specific habitats: in a flood meadow in Rusnė (site 6; meadows overgrown by reeds with the main vegetation being Poaceae and Cyperaceae plants) and in Žalgiriai forest (site 7; the main habitat spring-flooded block alder stands) (Table 1). Eastern Lithuania represented continental habitats (sites 8–12, Fig. 4). Site 8 was located in deciduous forest on a peninsula in the northern part of Lake Lukstas; site 9 in mixed forest-meadow ecotone; sites 10–11 in mixed forest and site 12 in deciduous forest on an island in Elektrėnai Reservoir (Table 1).

The dominant rodent species trapped in the western part of the country was *A*. *flavicollis* (n = 173), while in the eastern part of Lithuania, it was *M*. *glareolus* (n = 99). The indoor rodent species *M*. *musculus* was captured only in one sampling location (site 9) in the mixed forest-meadow ecotone in the continental area (Table 1). The highest rodent species diversity (six rodent species) was detected in the flooded forest (site 7) in the coastal area of the Nemunas River Delta. In contrast, only one rodent species, *M*. *glareolus* was detected in the deciduous forest on the island in Elektrėnai Reservoir (continental area; site 12) (Table 1).

Spleens from rodents were collected and stored in 70% ethanol for DNA extraction.

### Molecular analyses

DNA was extracted from each rodent spleen using a Genomic DNA Purification Kit, (Thermo Fisher Scientific, Lithuania), according to the manufacturer’s instructions. Screening for the presence of *Bartonella* DNA in rodents was conducted through the amplification of the 124-bp fragments of *ssrA* gene by TaqMan real-time PCR with ssrA-F1 and ssrA-R1 primers and ssrA-P1 probe (Table 3). The ssrA-F1 primer was designed in this study, while ssrA-R1 primer and ssrA-P1 hydrolyzation probe were obtained after modification of the reverse primer and hydrolyzation probe published in Diaz *et al*.^23^ on the basis of NCBI (National Center for Biotechnology Information) GenBank database sequences, NCBI BLAST® blastn suite applet for alignment and FastPCR online (http://primerdigital.com/tools/pcr.html) java applet for primers test. The qPCR reactions were carried out with SensiMix™ II Probe Kit (Bioline Reagents Ltd, UK) in a total volume of 15 μL using 1 μL of the isolated DNA sample, primers at a concentration of 200 nM each and probe at a concentration of 100 nM. Real-time PCR assays were performed employing a real-time thermocycler Rotor-Gene Q 5plex model with software version 1.7 (Qiagen GmbH, Germany) under the following conditions: denaturation at 95 °C for 10 min (1 cycle); followed by 50 cycles of denaturation at 95 °C for 20 s, annealing at 50 °C for 1 min and extension at 72 °C for 10 s. In each real-time PCR run, positive (DNA of *Bartonella*-infected rodents, confirmed by sequencing) and negative controls (which consisted of sterile, double-distilled water added to the PCR mix rather than DNA) were used. The results that satisfy amplification cutoffs below 40 C_t_ (cycle threshold) when threshold was 0.10101 indicated positive samples. Samples with C_t_ value below 30 were chosen for further molecular characterization of bacteria strains. For the amplifications of the 795 bp fragment of the RNA polymerase β-subunit (*rpoB*) gene and 336 bp of 60 kDa heat-shock protein (*groEL*), conventional PCR was used and for the amplification of 0.9–1.6 kb fragment of the 16S-23S rRNA gene intergenic species region (ITS), nested PCR was used (Table 3). A selected number of *Bartonella*-positive samples for two housekeeping genes (*rpoB*, *groEL*) and the ITS region (from different rodent species and different regions) were chosen for sequencing (Macrogen Europe, Netherlands). PCR products were extracted from agarose gel and purified using a GenJET PCR purification kit (Thermo Fisher Scientific, Lithuania). The obtained partial *rpoB*, *groEL* genes and ITS region sequences were analyzed using the Mega software package, version 6.05, and compared with the sequence data available from the NCBI GenBank database using the NCBI BLAST® blastn suite applet. Phylogenetic trees were constructed using the maximum-likelihood (ML) method with Tamura-Nei model.

**Table 3:** Oligonucleotide primers used for conventional, nested-PCR and real-time PCR of *ssrA*, *rpoB*, *groEL* genes and ITS region.

Primer	Locus	Nucleotide sequence	Reference
ssrA-R1	*ssrA*	AAGGCTTCTGTTGCCAGGYG	This study
ssrA-F1		AGTTGCAAATGACAACTATGCGG	
ssrA-P1^a^		FAM-ACCCCGCTTAA ACCTGCGACGGTT	
RpoB-F	*rpoB*	CGCATTGGYTTRCTTCGTATG	Renesto *et al*.^24^
RpoB-R		GTRGAYTGATTRGAACGYTG	
BTNgroEL1	*groEL*	GAAGATGTGGAAGGTGAA	
BTNgroEL2		TCACGGTCATAGTCAGAAG	
WITS-F^b^	ITS	ACCTCCTTTCTAAGGATGAT	Jensen *et al*.^25^; Kaewmongkol^26^
WITS-R^b^		CTCTTTCTTCAGATGATGATCC	
Bh311-332F^c^		CTCTTTCTTCAGATGATGATCC	
Bh473-452R^c^		AACCAACTGAGCTACAAGCCCT	
Inner ITS-R (ITS)^c^		GCGGTTAAGCTTCCAATCATA	

^a^Probe, ^b^External primers, ^c^Internal primers.

Partial *rpoB*, *groEL* genes and ITS region sequences for representative samples were submitted to the GenBank under the accession numbers MH547308 to MH547335 and MH687373 for the *rpoB* gene, MH547336 to MH547350 and MH687377-MH687379 for the ITS region, and MH547351 to MH547354 and MH687374-MH687376 for the *groEL* gene.

### Statistical analysis

Differences in the prevalence of *Bartonella* spp. infection between different species of small rodents, sampling locations, regions were assessed by Fisher’s exact test, supplemented with the Mantel-Haenszel common odds ratio estimate and 95% confidence intervals using SPSS software version 22 (IBM SPSS, Chicago, IL, USA). p < 0.05 was considered significant.

## References

[1] Birtles RJ, Raoult D (1996). Comparison of partial citrate synthase gene (*gltA*) sequences for phylogenetic analysis of *Bartonella* Species. Int. J. Syst. Bacteriol..

[2] Kosoy M (2010). Identification of *Bartonella* infections in febrile human patients from Thailand and their potential animal reservoirs. Am. J. Trop. Med. Hyg..

[3] Jiypong T, Jittapalapong S, Morand S, Rolain JM (2014). *Bartonella* species in small mammals and their potential vectors in Asia. Asian Pac J Trop Biomed.

[4] Okaro U, Addisu A, Casanas B, Anderson B (2017). *Bartonella* Species, an Emerging Cause of Blood-Culture-Negative Endocarditis. Clin Microbiol Rev.

[5] Buffet JP, Kosoy M, Vayssier-Taussat M (2013). Natural history of *Bartonella*-inceting rodents in light of new knowledge on genomics, diversity and evolution. Future Microbiol.

[6] Mullins KE (2017). Whole-genome analysis of *Bartonella* ancashensis, a novel pathogen causing verruga peruana, rural Ancash region, Peru. Emerging Infect. Dis..

[7] Goncalves LR (2016). Association of *Bartonella* species with wild and synanthropic rodents in different Brazilian biomes. Appl. Environ. Microbiol..

[8] Gutierrez R (2015). *Bartonella* infection in rodents and their flea ectoparasites: an overview. Vector Borne Zoonotic Dis..

[9] Morick D, Krasnov BR, Khokhlova IS, Gottlieb Y, Harrus S (2011). Investigation of *Bartonella* acquisition and transmission in *Xenopsylla ramesis* fleas (Siphonaptera: Pulicidae). Mol. Ecol..

[10] Silaghi C, Pfeffer M, Kiefer M, Obiegala A (2016). *Bartonella*, rodents, fleas and ticks: a molecular field study on host-vector-pathogen associations in Saxony, Eastern Germany. Microb. Ecol..

[11] Špitalska E (2017). Diversity and prevalence of *Bartonella* species in small mammals from Slovakia, Central Europe. Parasitol. Res..

[12] Knap N (2007). Molecular detection of *Bartonella* species infecting rodents in Slovenia. FEMS Immunol. Med. Microbiol..

[13] Engbaek K, Lawson PA (2004). Identification of *Bartonella* species in rodents, shrews and cats in Denmark: Detection of two *B*. *henselae* variants, one in cats and the other in the long-tailed field mouse. APMIS.

[14] Welc-Faleciak R, Paziewska A, Bajer A, Behnke JM, Sinski E (2008). *Bartonella* spp. infection in rodents from different habitats in Mazury Lake District, Northeast Poland. Vector Borne Zoonotic Dis..

[15] Holmeberg M, Mills JN, McGill S, Benjamin G, Ellis BA (2003). *Bartonella* infection in sylvatic small mammals of central Sweden. Epidemiol. Infect..

[16] Buffet JP (2012). Co-infection of *Borrelia afzelii* and *Bartonella* spp. in bank voles from a suburban forest. Comp. Immunol. Microbiol. Infect. Dis..

[17] Buffet JP (2013). Deciphering *Bartonella* diversity, recombination, and host specificity in a rodent community. PLoS One.

[18] Eremeeva ME (2007). Bacteremia, fever, and splenomegaly caused by a newly recognized *Bartonella* species. N. Engl. J. Med..

[19] Kraljik J (2016). Genetic diversity of *Bartonella* genotypes found in the striped field mouse (*Apodemus agrarius*) in Central Europe. Parasitology..

[20] Lipatova I (2015). *Bartonella* infection in small mammals and their ectoparasites in Lithuania. Microbes Infect.

[21] Ko S (2016). Prevalence, isolation and molecular characterization of *Bartonella* species in Republic of Korea. Transbound Emerg Dis.

[22] Zalakevicius M, Stanevicius V, Svazas S, Bartkevicienė G (2012). The importance of potential impact of climate change on bird species composition in designing effective ways of bird protection and management: a case study from the eastern Baltic region. J Environ Eng Landsc..

[23] Diaz MH, Bai Y, Malania L, Winchell JM, Kosoyb MY (2012). Development of a novel genus-specific real-time PCR assay for detection and differentiation of *Bartonella* species and genotypes. J. Clin. Microbiol..

[24] Renesto P, Gouvernet J, Drancourt M, Roux V, Raoult D (2001). Use of *rpoB* gene analysis for detection and identification of *Bartonella* species. J. Clin. Microbiol..

[25] Jensen WA, Fall MZ, Rooney J, Kordick DL, Breitschwerdt EB (2000). Rapid identification and differentiation of *Bartonella* species using a single-step PCR Assay. J. Clin. Microbiol..

[26] Kaewmongkol, G. Detection and characterization of *Bartonella* species in Western Australia (Thesis of Doctor of Philosophy). School of Veterinary and Biomedical Sciences, Faculty of Health Sciences, Murdoch University, Perth, Western Australia (2012).

